# Supplementary material for: A molecular vision of fungal cell wall organization by functional genomics and solid-state NMR
Source: Nat Commun. 2021 Nov 3;12:6346. doi: 10.1038/s41467-021-26749-z (PMC8566572; doi:10.1038/s41467-021-26749-z)
Supplement: Supplementary file 1 — Supplementary Information [file 41467_2021_26749_MOESM1_ESM.pdf]

Supplementary Information

A Molecular Vision of Fungal Cell Wall Organization by Functional Genomics and Solid-State NMR

Arnab Chakraborty[1,#], Liyanage D. Fernando[1,#], Wenxia Fang[2,#], Malitha C. Dickwella Widanage[1,#], Pingzhen Wei[2], Cheng Jin[2,3], Thierry Fontaine[4], Jean-Paul Latgé[5,*], Tuo Wang[1,*]

[1] Department of Chemistry, Louisiana State University, Baton Rouge, LA, United States
[2] State Key Laboratory of Non-food Biomass and Enzyme Technology, Guangxi Academy of Sciences, Nanning, China
[3] State Key Laboratory of Mycology, Institute of Microbiology, Chinese Academy of Sciences, Beijing, China
[4] Unité de Biologie et pathogénicité fongiques, INRAE, USC2019, Institut Pasteur, Paris, France
[5] Institute of Molecular biology and Biotechnology (IMBBFORTH), University of Crete, Heraklion, Greece

[#]These authors contributed equally to this work

* Correspondence and requests for materials should be addressed to T.W. and J.-P. L.

(Email: tuowang@lsu.edu; jean-paul.latge@pasteur.fr)

Table of Content

Supplementary Methods 3

Supplementary Figure 1. Differentiation of the rigid and mobile molecules 5

Supplementary Figure 2. Reproducibility evidenced by 1D ^{13}C spectra 6

Supplementary Figure 3. Comparison of cell walls of different mutants 7

Supplementary Figure 4. Distribution of FWHM linewidths 8

Supplementary Figure 5. Mobile polysaccharides of fungal cell walls 9

Supplementary Figure 6. NaOH treated alkali-insoluble fraction 10

Supplementary Figure 7. Proteins in alkali-soluble fraction 11

Supplementary Figure 8. Overlay of 2D ^{13}C spectra of whole cells and alkali extracts 12

Supplementary Figure 9. Mobile proteins in *A. fumigatus* 13

Supplementary Figure 10. ^{13}C-T_1 relaxation measurements 14

Supplementary Figure 11. Water-to-polysaccharide buildup curves 15

Supplementary Figure 12. Distribution of cell wall thickness 16

Supplementary Table 1. Parameters of ssNMR experiments 17

Supplementary Table 2. Chemical shifts of polysaccharides and proteins 18

Supplementary Table 3. Compositional change of rigid polysaccharides 20

Supplementary Table 4. Compositional changes in the mobile polysaccharides 21

Supplementary Table 5: NMR peaks used for compositional analysis 22

Supplementary Table 6. Polysaccharide composition from ssNMR data 23

Supplementary Table 7. Chemical analysis of *A. fumigatus* polysaccharides 24

Supplementary Table 8. ^{13}C-T_1 relaxation times of polysaccharides 25

Supplementary Table 9. Water-edited buildup curves of polysaccharides 26

Supplementary References 28

Supplementary Methods

Composition of minimum media for isotopically labeling fungal cells. To obtain isotopically labeled fungal cells, minimum media were prepared with the following components: 1% ^{13}C-glucose, 0.6% ^{15}N-$NaNO_3$, 1 mL/L of 1000X trace elements, 20 mL/L 50X salt solution, 50 mM Mops to adjust the final pH to 7. 1000X trace elements solution was composed of 0.04 ‰ $Na_2B_4O_7 \cdot 10H_2O$, 0.4 ‰ $CuSO_4 \cdot 5H_2O$, 0.8 ‰ $MnSO_4 \cdot 4H_2O$, 0.8 ‰ $Na_2MoO_4 \cdot 10H_2O$, 8 ‰ $ZnSO_4 \cdot 7H_2O$, 5 mM $FeCl_3$, and 0.2 M HCl to prevent oxidation. 50X salt solution is composed of 26‰ KCl, 26‰ $MgSO_4 \cdot 7H_2O$, and 76‰ KH_2PO_4.

Transmission electron microscope measurement. In total, 3 μL of each sample was placed onto a glow discharged TEM grid for several minutes and stained using a mixture of 2% uranyl acetate and lead citrate solution. A thin film was spanned on the grid by removing the excess solution with the paper filter. The TEM images were collected using a JEOL JEM-1400 electron microscope. Cell wall thickness was measured using ImageJ software after setting the scale in accordance with known bar scales on the cell images. Statistical analysis was done using unpaired student's t-test for all the mutant samples on the cell wall thickness measurements performed.

Estimation of carbohydrate composition using resolved NMR signals. To estimate the amount of different polysaccharides, we used 2D 53-ms CORD and DP J-INADEQUATE spectra for rigid and mobile phases, respectively. The peak volumes in the 2D spectra are obtained using the integration function of the Bruker Topspin software. The assignment of the cross peaks and the peak volumes are provided in the Source Data as well as the descriptions associated with **Supplementary Tables 3** and **4**. To minimize the effect from spectral overlapping in the CORD spectrum (which has diagonal), we typically avoid considering the cross peaks involving any of the heavily overlapped carbon sites such as the C6 position of β-1,3-glucan, the chitin C1, unless a resolved cross peak is present. For the closely placed signals of the C2 (71.9 ppm) and C4 (71.7 ppm) of the α-1,3-glucan, we divided the sum of their peak integral equally. For the diagonal-free INADEQUATE spectrum: we mostly rely on the resolved C1-C2 and C4-C5 spin connections. The NMR peaks used for quantification are provided in **Supplementary Table 5** and Source Data file.

The relative abundance of a specific polysaccharide ($\mathrm{RA}^{\mathrm{poly.}x}$) was calculated by normalizing the sum of integrals by the number of peaks using the following equation:

$$\mathrm{RA}^{\mathrm{poly.}x}(\%) = \frac{\sum_{n=1}^{n_{\mathrm{peaks}}^{\mathrm{poly.}x}} \mathrm{I}_n^{\mathrm{poly.}x} \,/\, n_{\mathrm{peaks}}^{\mathrm{poly.}x}}{\sum_{m=1}^{m^{\mathrm{poly.}}} \left(\sum_{n=1}^{n_{\mathrm{peaks}}^{\mathrm{poly.}x}} \mathrm{I}_n^{\mathrm{poly.}x} \,/\, n_{\mathrm{peaks}}^{\mathrm{poly.}x}\right)} \times 100$$

where $n_{\mathrm{peaks}}^{\mathrm{poly.}x}$ is the number of cross-peaks, $\mathrm{I}_n^{\mathrm{poly.}x}$ is the integral (peak volume), and $m^{\mathrm{poly.}}$ is the total number of cell wall polysaccharides.

The Error was determined by calculating the standard error of a specific polysaccharide ($\mathrm{std.ERR}^{\mathrm{poly.}x}$) using the standard deviation of integrated peak volume dividing by square summation of the number of cross-peaks. The total standard error ($\sum \mathrm{std.ERR}$) calculated by the square sum of the standard error of each polysaccharide. Finally, percentage error of specific polysaccharide ($\mathrm{ERR}^{\mathrm{poly.}x}$) calculated by the fraction of standard error ($\mathrm{std.ERR}^{\mathrm{poly.}x}$) in average integrated peak volume of specific polysaccharide ($\overline{x}^{V^{\mathrm{poly.}x}}$) and the fraction of total standard error ($\sum \mathrm{std.ERR}$) in total integrated peak volume ($\sum V^{\mathrm{poly.}}$) followed by multiplication with the relative abundance of the specific polysaccharide ($\mathrm{RA}^{\mathrm{poly.}x}$). The process can be presented using the following equation:

$$\mathrm{ERR}^{\mathrm{poly.}x} = \frac{\mathrm{std.ERR}^{\mathrm{poly.}x}}{\overline{x}^{V^{\mathrm{poly.}x}}} \times \frac{\sum \mathrm{std.ERR}}{\sum V^{\mathrm{poly.}}} \times \mathrm{RA}^{\mathrm{poly.}x}$$

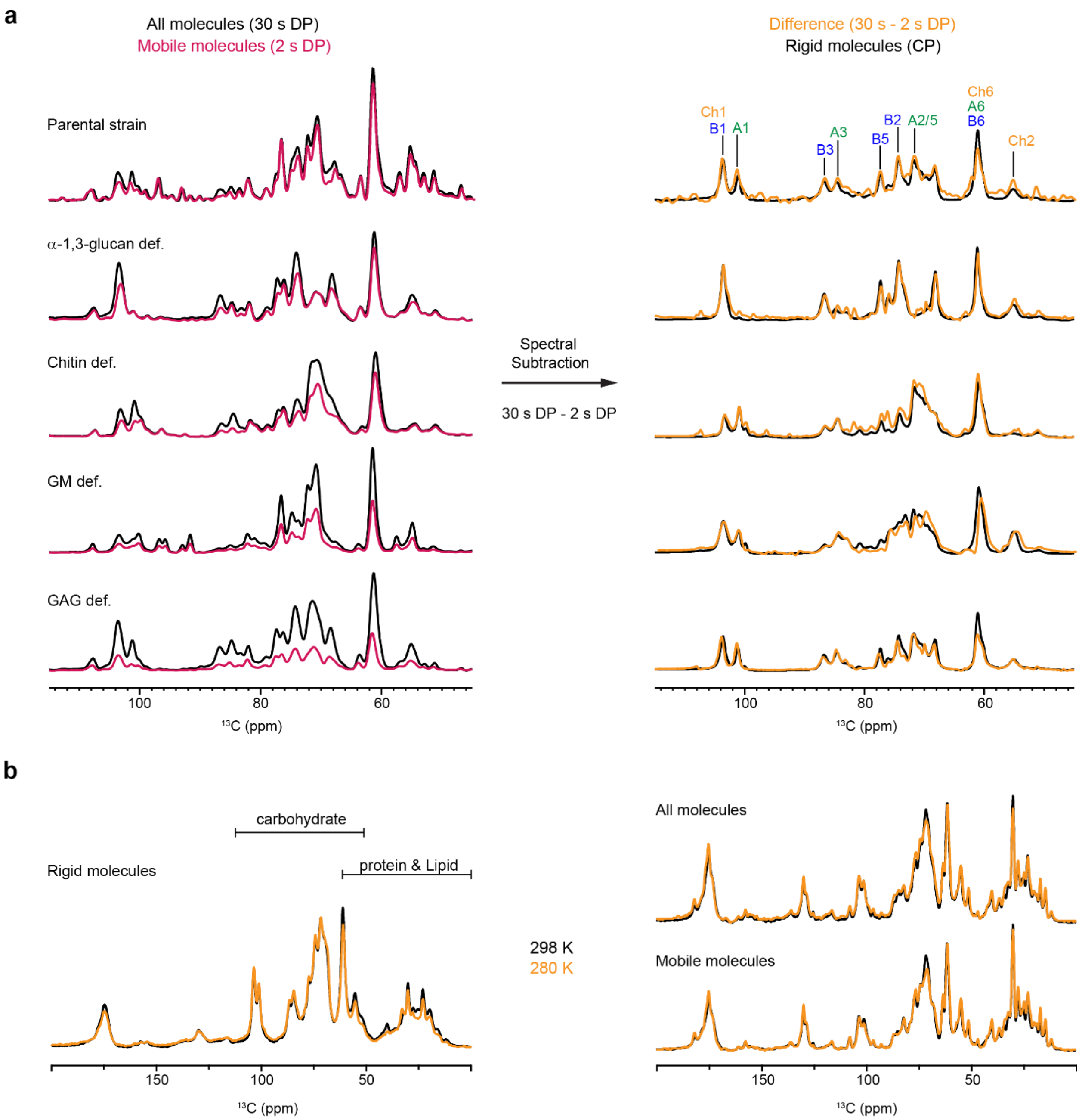

Supplementary Figure 1. Differentiation of the rigid and mobile molecules in *A. fumigatus*. a, Overlay of 1D ^{13}C DP spectra measured using recycle delays of 30 s (black spectrum; quantitatively detecting all molecules) and 2 s (magenta spectrum, selectively detecting the mobile molecules). For each sample, subtraction of the two DP spectra generates a difference spectrum (yellow) reporting the rigid molecules. The patterns of the difference spectra are similar to those of the CP spectra. Therefore, the use of CP and DP with short recycle delays and CP in 2D spectra will efficiently probe most molecules in the sample. **b**, 1D ^{13}C CP and DP spectra of the parental strain measured at 298 K and 280 K. The difference in the pattern of carbohydrate signals at these two temperatures is negligible.

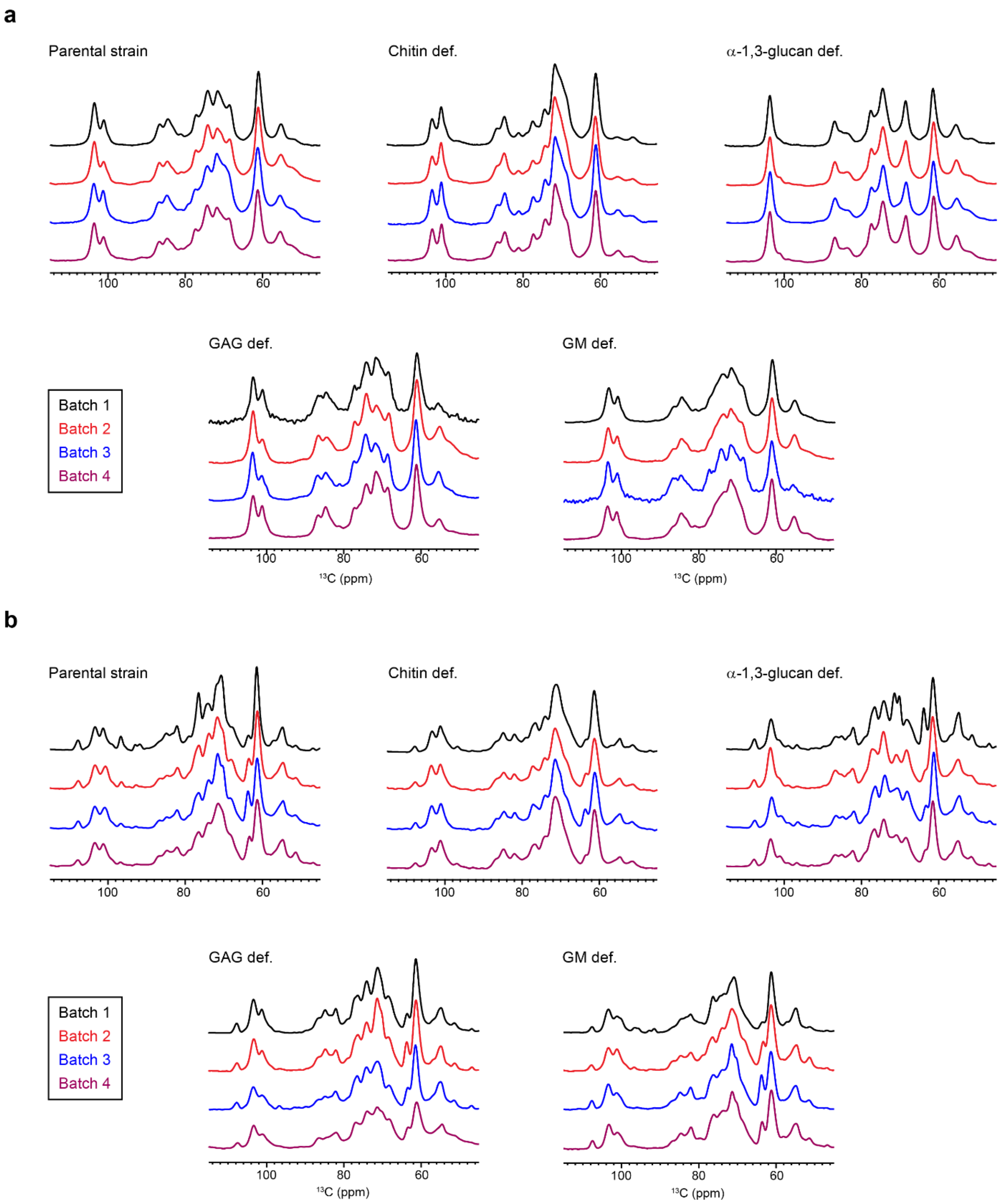

Supplementary Figure 2. Reproducibility evidenced by 1D ^{13}C spectra of four batches of *A. fumigatus* samples. Four ^{13}C,^{15}N-labeled samples were prepared for each of the five strains. Batch 1 was the original sample used for the majority of the research described in this study, and batch 2-4 were prepared freshly for reproducibility test. **a**, 1D ^{13}C CP detecting the rigid molecules. **b**, 1D quantitative DP spectra collected using a long recycle delay of 30 s for the unbiased detection of all molecules.

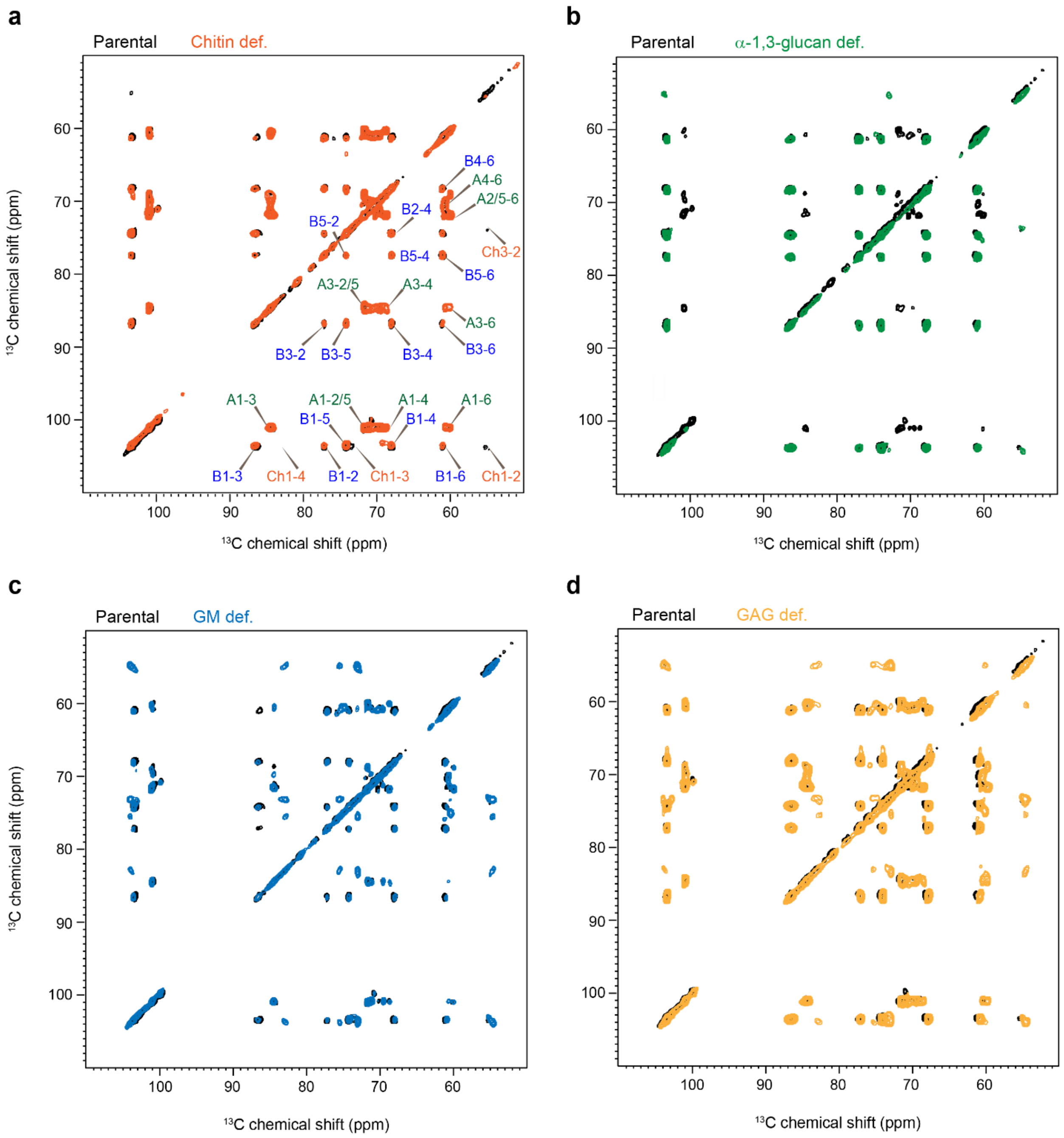

Supplementary Figure 3. Comparison of cell walls of different mutants of *A. fumigatus*. The overlay of 2D ^{13}C-^{13}C CORD spectra of wild-type sample (black) with **a**, chitin-deficient mutant (orange), **b**, α-1,3-glucan-deficient strain (green), **c**, galactomannan-deficient mutant (cyan), and **d**, galactosaminogalactan-deficient mutant (yellow). These spectra selectively detect rigid molecules including chitin, α-1,3-glucan, and β-glucan. Signals of either chitin or α-1,3-glucan are missing in the corresponding mutants. The galactomannan-deficient and galactosaminogalactan-deficient mutants do not miss any signals. The galactomannan-deficient sample has a higher amount of chitin. All the spectra were measured on an 800 MHz spectrometer under 13 kHz MAS.

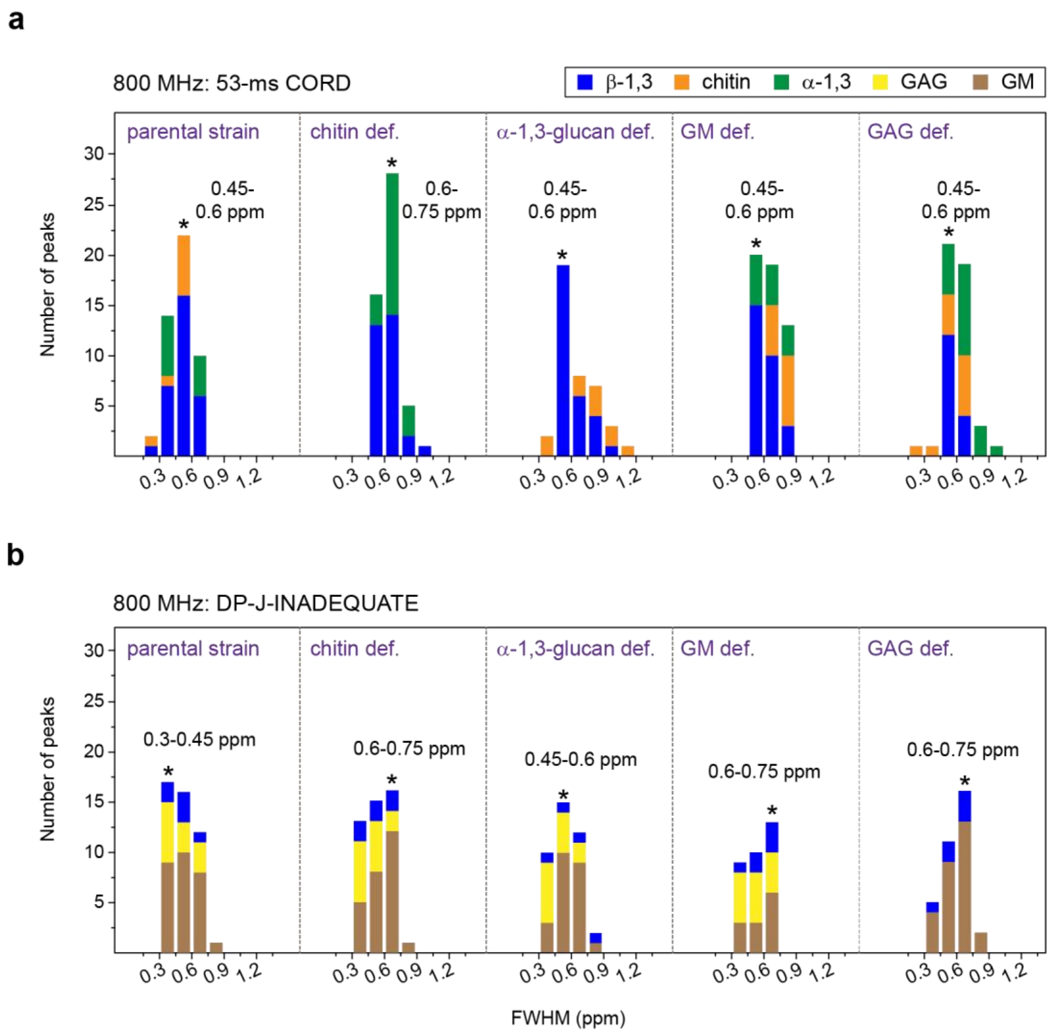

Supplementary Figure 4. Distribution of FWHM linewidth. a, Spectral linewidth of ^{13}C whole cell sample centered at 0.45-0.75 ppm region for the 53 ms CORD spectra collected on an 800 MHz NMR. **b**, FWHM linewidth of DP J-INADEQUATE spectra clustered in the 0.3-0.75 ppm region. The data shown are whole-cell samples measured on an 800 MHz spectrometer. Source data are provided as a Source Data file.

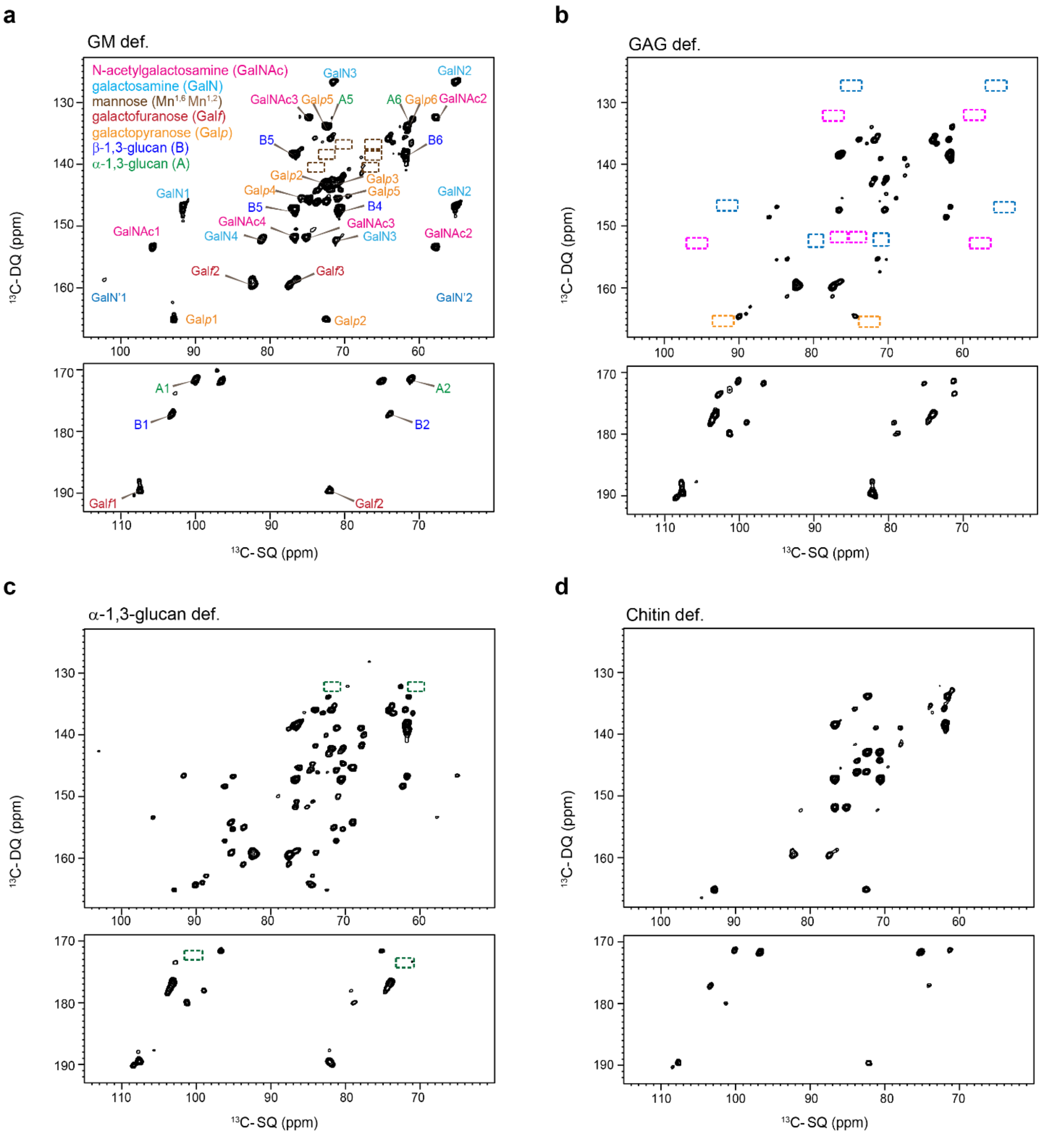

Supplementary Figure 5. Mobile Polysaccharides of fungal cell walls. 2D ^{13}C DP J-INADEQUATE spectra are compared among **a**, GM-deficient mutant, **b**, GAG-deficient sample, **c**, α-1,3-glucan-deficient strain, and **d**, chitin-deficient sample. The use of ^{13}C-DP and short recycle delays selectively probes the rigid domains of polysaccharides. Dashline boxes are used to indicate the absence of mannose signals in the GM-deficient mutant, the absence of GalNAc and $GalNH_2$ peaks in the GAG-deficient sample, as well as the missing α-1,3-glucan regions in the α-1,3-glucan-deficient mutant. Chitin is not present in the mobile region hence no missing peaks were observed in the chitin-deficient sample.

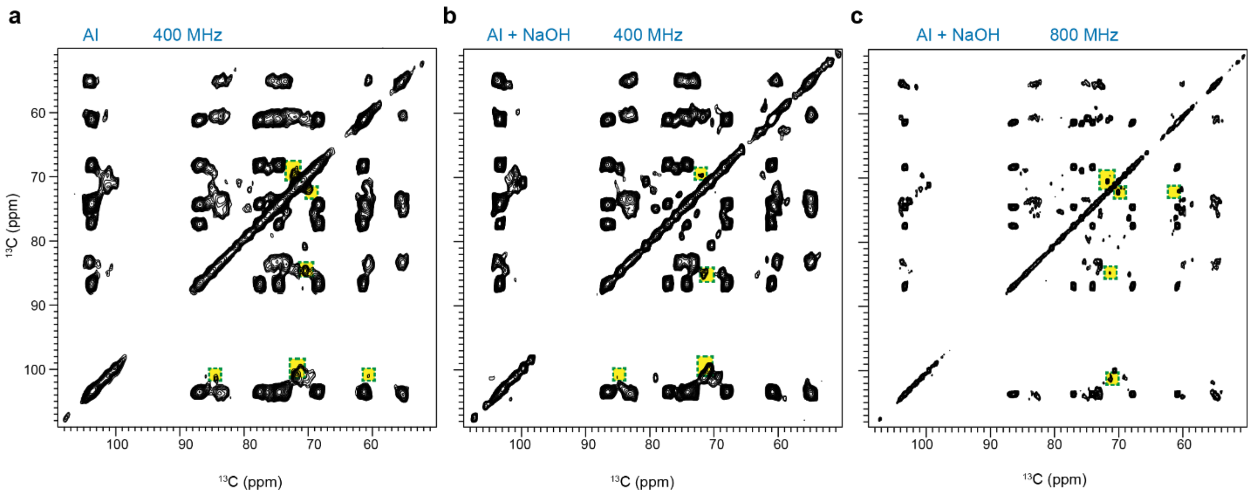

Supplementary Figure 6. NaOH treated alkali-insoluble fraction. ^{13}C-^{13}C 2D CORD spectra of the alkali-insoluble (AI) fraction **a**, before a second NaOH treatment, and **b**, after a second NaOH at a 400 MHz spectrometer. **c**, AI portion treated again with NaOH and measured on an 800 MHz spectrometer, which shows much narrower peaks as benefited from the resolution improvement. Highlighted regions show the α-1,3-glucan signals, which were retained after multiple times of NaOH treatments.

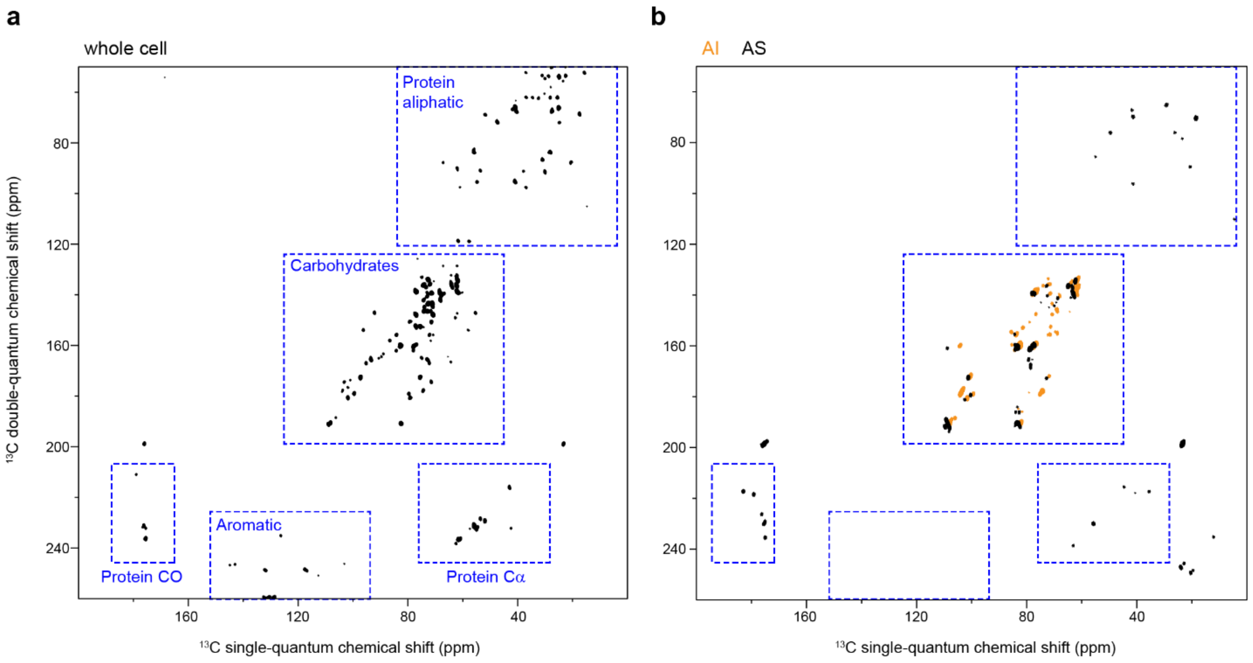

Supplementary Figure 7. Presence of proteins in alkali-soluble fraction. a, Full DP J-INADEQUATE spectra of parental *A. fumigatus* cells detecting mobile molecules. **b**, Overlay of DP J-INADEQUATE spectra collected on the alkali-insoluble (AI; yellow) and alkali-soluble (AS) fractions of *A. fumigatus* cell walls. Protein signals are mainly identified in the mobile part of the AS fraction.

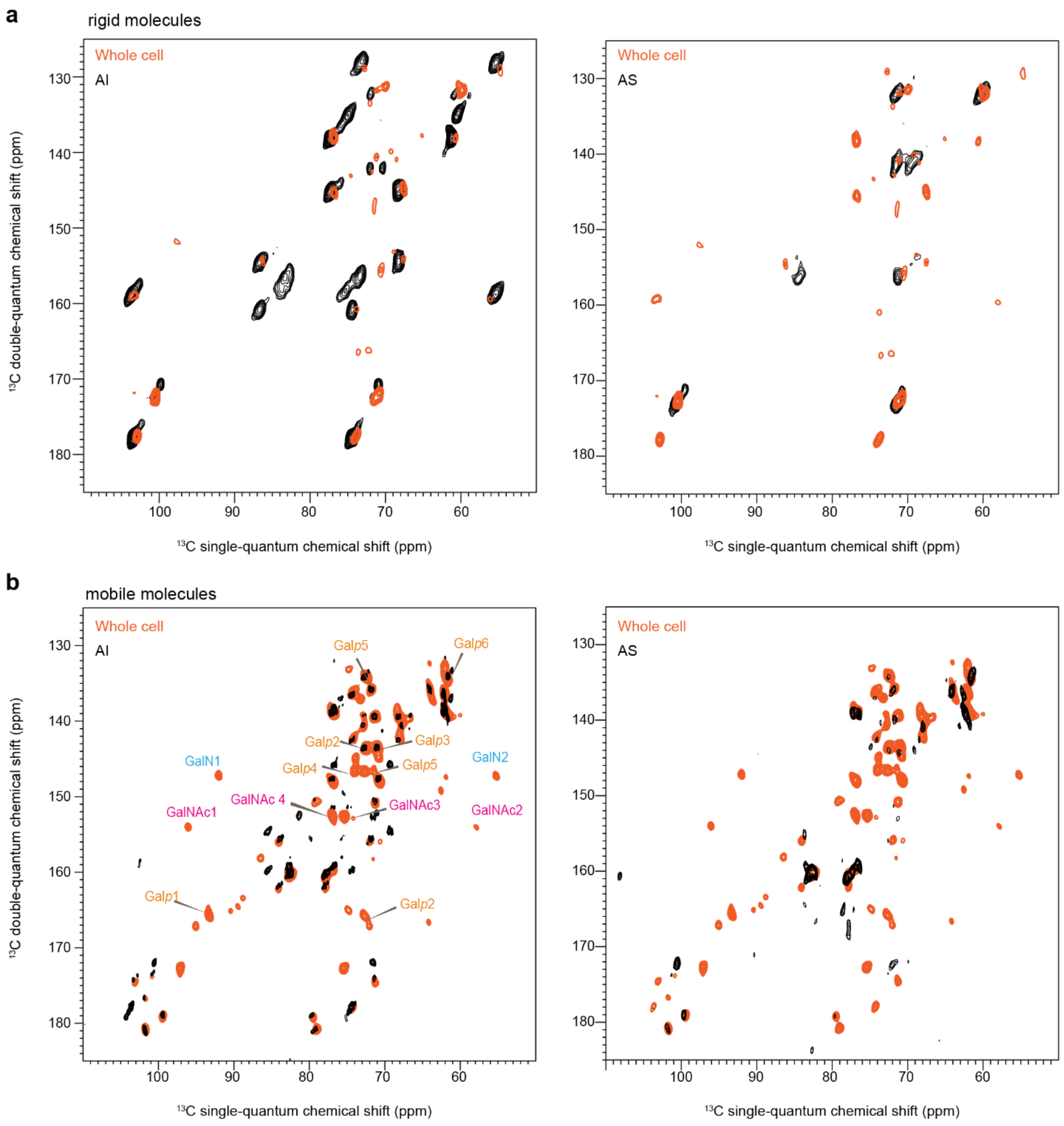

Supplementary Figure 8. Overlay of 2D ^{13}C spectra of whole cells and alkali extracts. a, ^{13}C CP INADEQUATE spectra of wild-type intact cells and the alkali-insoluble (AI) and alkali-soluble (AS) samples, showing signals of rigid molecules These spectra preferentially detect rigid molecules. **b**, DP J-INADEQUATE spectra of the whole cell of the parental strain, as well as the AI and AS samples, detecting only the mobile molecules.

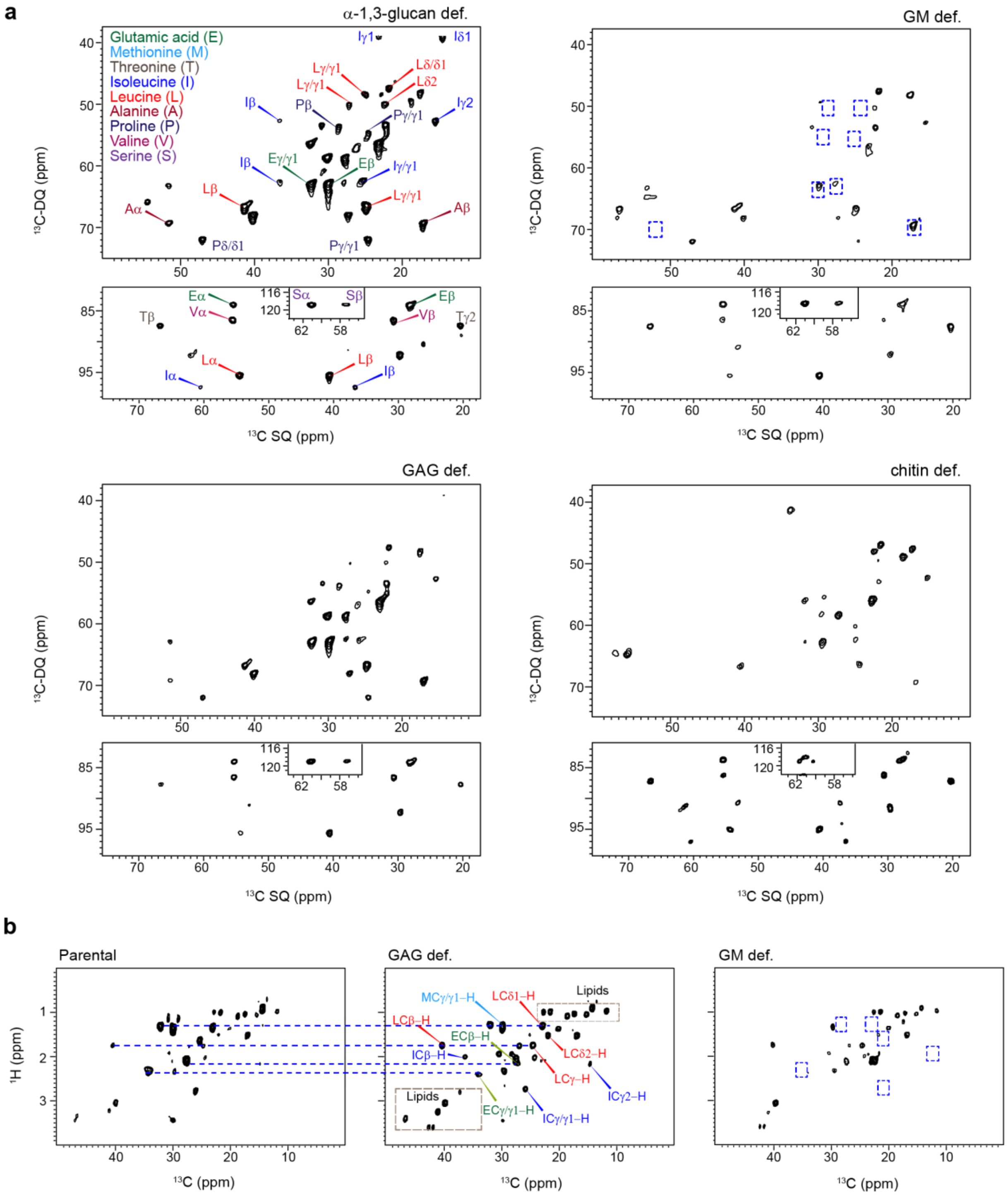

Supplementary Figure 9. Mobile proteins in *A. fumigatus*. **a**, Protein regions of ^{13}C DP J-INADEQUATE spectra that probe mobile molecules. GM-deficient sample is missing most amino acid signals (blue boxes). **b**, 2D ^{1}H-^{13}C INEPT spectra reinforcing that the GM-deficient mutant lacks many protein signals.

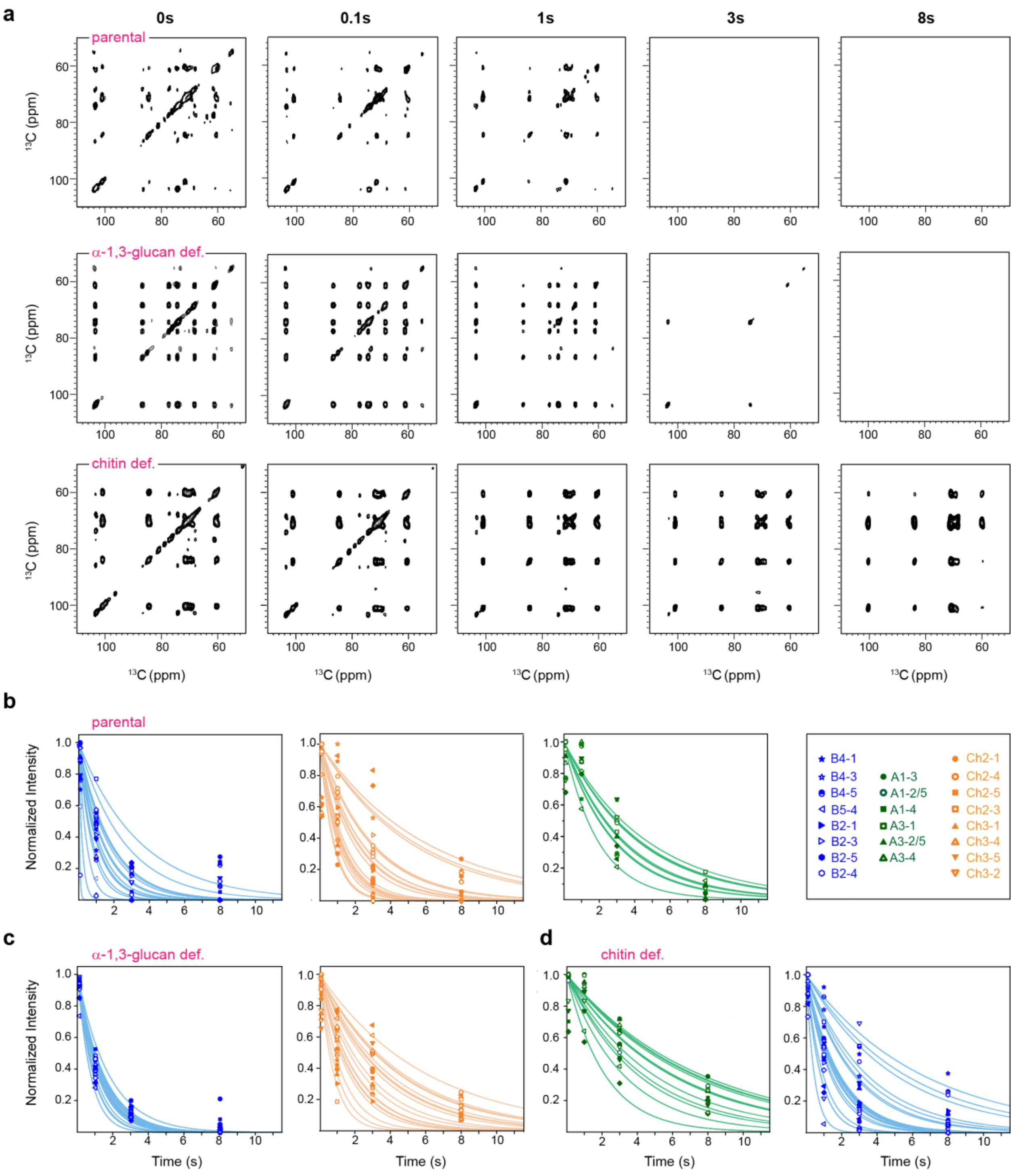

Supplementary Figure 10. ^{13}C-T$_1$ relaxation measurements of *A. fumigatus*. a, 2D ^{13}C-^{13}C spectra with a variable z-filter collected on the parental strain (top), α-1,3-glucan-deficient (middle), and chitin-deficient samples (bottom). Within each sample, 5 representative spectra are shown with different z-filter times of 0s, 0.1s, 1 s, 3s, and 8s. ^{13}C-T$_1$ relaxation curves of polysaccharides of **b**, parental strain, **c**, a-1,3-glucan deficient strain and **d**, chitin-deficient sample were obtained by plotting the normalized intensities as a function of time. Data were collected on a 400 MHz spectrometer and best-fits were obtained using a single exponential equation. Source data are provided as a Source Data file.

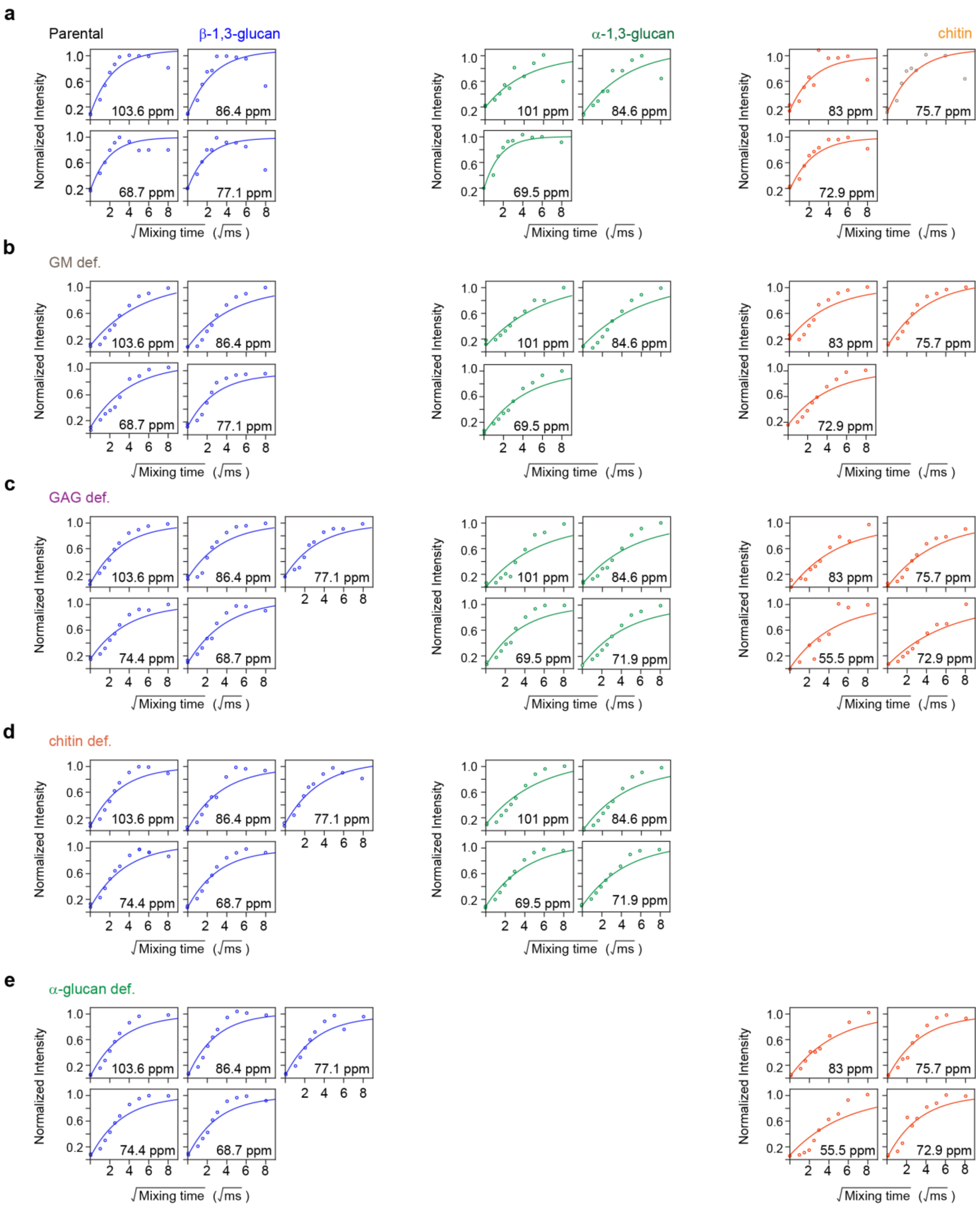

Supplementary Figure 11. Water-to-polysaccharide buildup curves. The data are plotted separately for **a**, wild-type sample, **b**, GM-deficient mutant, **c**, GAG-deficient strain, **d**, chitin-deficient sample, and **e**, α-1,3-glucan deficient mutant. Source data are provided as a Source Data file.

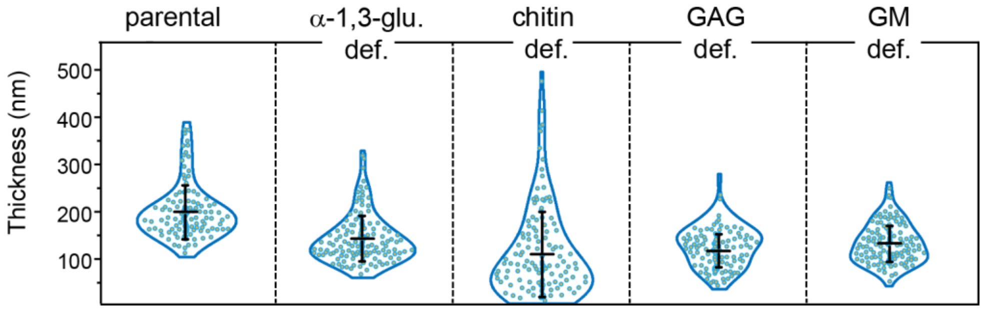

Supplementary Figure 12. Distribution of cell wall thickness. Violin plots showing the distribution of cell wall thickness measured using TEM of parental, chitin-deficient, α-1,3-glucan-deficient, GM-deficient, and GAG-deficient cell walls (n=143). Source data are provided as a Source Data file.

Supplementary Table 1. Parameters of ssNMR experiments measured on each *A. fumigatus* strain. All the experiments were conducted on each of the five fungal strains, mostly on an 800 MHz NMR spectrometer. In addition, the alkaline soluble and insoluble samples were measured on a 400 MHz spectrometer. The key experimental parameters listed here include recycle delay (d1), number of scans (NS), number of points for the direct (td2) and indirect (td1) dimensions, the acquisition time of the direct dimension (aq2) and the evolution time of indirect dimension (aq1), ^{13}C-^{13}C or ^{1}H-^{1}H mixing time (t_m), z-filter time (t_z), and ^{1}H Larmor frequency.

	Expeiment	d1 (s)	NS	td2	td1	aq2 (ms)	aq1 (ms)	t_m (ms)	t_z(s)	υ_0, ^{1}H (MHz)
1D	CP	2	128	2400	1	16.8				800
	DP	2	128	4096	1	28.7				
		30	64	4096	1	28.7				
	Water-edited	1.8	2048	1400	1	14.0		0, 1, 2.25, 4, 6.25, 9, 16, 25, 36, 64		400
2D	CORD	2	32	2400	200	16.8	5.6	53		800
		2	128	1600	280	16.0	7.0	53		400
	N(CA)CX	1.7	256	2200	84	16.4	4.4	100		800
	DP-J-INDQUATE	2	8	2600	1024	19.4	10.2			
	CP-SPC-5-INDQUATE	2	16	2400	200	17.9	7.0			
	Pseudo 3D ^{13}C-T_1	1.6	64	1600	98	16	5.39		0, 0.1, 1, 3, 8	400
	INEPT	3	8	2048	160	20.5	11.0			

Supplementary Table 2. ^{13}C and ^{13}N chemical shifts of polysaccharides and proteins in *A. fumigatus* cell walls. Superscripts are used to denote different allomorphs. Underline denotes the ^{13}C connectivity with ambiguity. Weak signals or minor species are indicated using "w." Not applicable (/). Unidentified (-). Unk: unknown.

Biomolecule	C1	C2	C3	C4	C5	C6	CO	CH_3	N	Experiment	Cell wall portion	References
B	103.6	74.4	86.4	68.7	77.1	61.3	/	/	/	^{13}C -^{13}C PDSD, ^{13}C CP J-INADEQUATE	Rigid	Shim et al. 2007[1]<br>Fairweather et al. 2004[2]<br>Hazime Saitô et al. 1979[3]
B	103.3	74.2	88.3	70.1	77.0	61.2	/	/	/	^{13}C DP J-INADEQUATE	Mobile	Shim et al. 2007[1]<br>Fairweather et al. 2004[2]<br>Hazime Saitô et al. 1979[3]
A	101.0	71.9	84.6	69.5	71.7	60.5	/	/	/	^{13}C -^{13}C PDSD, ^{13}C CP J-INADEQUATE	Rigid	Bhanja et al. 2014[4]<br>Puanglek et al. 2016[5]
Ch	103.6	55.5	72.9	83.0	75.7	60.9	174.8	22.6	123.6	^{13}C -^{13}C PDSD, ^{13}C CP J-INADEQUATE, ^{15}N -^{13}C N(CA)CX-DARR	Rigid	Kono et al. 2004[6]<br>Heux et al. 2000[7]<br>Kameda et al. 2004[8]<br>King et al. 2017[9]<br>Tanner 1990[10]
$Mn^{1,2}$	101.3	78.7	71.2	67.7	73.9	61.7		/		^{13}C DP J-INADEQUATE	Mobile	Latge et al.1994[11]
$Mn^{1,6}$	102.7	70.6	73.2	67.9	73.7	66.1		/		^{13}C DP J-INADEQUATE	Mobile	
Gal*f*	107.5	81.6	77.7	83.5	71.5	63.5		/		^{13}C DP J-INADEQUATE	Mobile	
Gal*p*	93.2	72.2	70.7	73.5	72.5	60.9		/		^{13}C DP J-INADEQUATE	Mobile	Fontaine et al. 2011[12]
GalN	91.7	54.8	71.1	81.1	/	/		/	/	^{13}C DP J-INADEQUATE	Mobile	
GalN'/ GalNAc'	102.5	55.8	71.1	83.6	/	/		/	/	^{13}C DP J-INADEQUATE	Mobile	
GalNAc	95.7	57.5	75.2	76.9	/	/	175.2	22.7	/	^{13}C DP J-INADEQUATE	Mobile	
Leucine	54.9	40.6	24.4	22.7			175.5	21.6	-	^{13}C DP J-INADEQUATE	Mobile	Fritzsching et al. 2013[13]
Leucine	55.1	/	/	22.7			175.6	21.7		^{13}C -^{13}C CORD	Rigid	
Isoleucine	60.7	36.4	25.2	11.9			175.2	15.8	-	^{13}C DP J-INADEQUATE	Mobile	
Alanine	52.1	16.7					176.5			^{13}C DP J-INADEQUATE	Mobile	

Serine	57.3	60.9					174.4			^{13}C DP J-INADEQUATE	Mobile	
Serine	59.2	62.7					174.8			^{13}C -^{13}C CORD	Rigid	
Glutamic acid	55.5	27.7	34.4	/	/	/	175.8	/	-	^{13}C DP J-INADEQUATE	Mobile	
Methionine	53.7	29.4	29.8	/	/	/	176.4	14.2	-	^{13}C DP J-INADEQUATE	Mobile	
Threonine	61.1	66.6	19.9	/	/	/	175.3	/	-	^{13}C DP J-INADEQUATE	Mobile	
Proline	61.3	27.1	24.2	46.8	/	/	174.7	/	-	^{13}C DP J-INADEQUATE	Mobile	
Argenine	55.2	27.9	24.7	25.9	40.9	/	175.8			^{13}C DP J-INADEQUATE	Mobile	
Tyrosine	53.9	34.3	129.3	/	/	115.7	172.9		128.7	^{13}C DP J-INADEQUATE	Mobile	Fritzsching et al. 2013[13]
Tyrosine	55.7	36.4	/	/	/	/	173.2		129.6	^{13}C -^{13}C CORD	Rigid	
Valine	60.8	29.5	17.3	19.5	/	/	173.8	18.6	128.7	^{13}C DP J-INADEQUATE	Mobile	
Valine	60.8	28.6	18.9	19.5			174	/	129	^{13}C -^{13}C CORD	Rigid	

Supplementary Table 3. Compositional change of rigid polysaccharides in different samples. UD: undetected. Error bars are standard errors of cross peak intensities.

Wild type

	β-1,3-glucan	Chitin	α-1,3-glucan
Percentage (mol%)	50±6	8±3	42±7

α–1,3-glucan def.

	β-1,3-glucan	Chitin	α-1,3-glucan
Percentage (mol%)	95±9	5.3±0.6	UD

Chitin def.

	β-1,3-glucan	Chitin	α-1,3-glucan
Percentage (mol%)	42±4	UD	58±5

GM def.

	β-1,3-glucan	Chitin	α-1,3-glucan
Percentage (mol%)	25±2	43±6	32±3

GAG def.

	β-1,3-glucan	Chitin	α-1,3-glucan
Percentage (mol%)	58±5	5.9±0.8	36±5

The areas of the following resolved cross peaks of 53ms CORD spectra are used for the calculation:
β-1,3-glucan: the average of C1- C3/C4/C5, C3-C2/C4/C5, C2-C4, C5-C4
α-1,3-glucan: the average of C1-C4/C2/C5, C3-C2/C4/C5
Chitin: the average of C1-C2/ C3/ C4/ C5, C4- C2/C3/C5, C5-C3, C3-C2, C5-C2

Supplementary Table 4. Compositional changes in the mobile polysaccharides of *A. fumigatus*. UD: undetected. Error bars are standard errors of cross peak intensities.

Parental

	GM			GAG			β-1,3-glucan	α-1,3-glucan
Percentage (mol%)	Gal*f*	$Mn^{1,2}$	$Mn^{1,6}$	Gal*p*	GalN	GalNAc	4±1	0.83±0.09
	20±2	24±1	5.1±0.3	27±2	13±3	6±1		

α–1,3-glucan def.

	GM			GAG			β-1,3-glucan	α-1,3-glucan
Percentage (mol%)	Gal*f*	$Mn^{1,2}$	$Mn^{1,6}$	Gal*p*	GalN	GalNAc	42±8	UD
	27±2	5±1	4±1	14±1	4.5±0.8	3.0±0.5		

Chitin def.

	GM			GAG			β-1,3-glucan	α-1,3-glucan
Percentage (mol%)	Gal*f*	$Mn^{1,2}$	$Mn^{1,6}$	Gal*p*	GalN	GalNAc	14±3	19±3
	20±3	9±1	7±1	28±4	1.3±0.4	2.0±0.4		

GM def.

	GM			GAG			β-1,3-glucan	α-1,3-glucan
Percentage (mol%)	Gal*f*	$Mn^{1,2}$	$Mn^{1,6}$	Gal*p*	GalN	GalNAc	5.8±0.9	10±1
	7.8±0.9	0.62±0.05	0.97±0.07	33±3	22±4	19±3		

GAG def.

	GM			GAG			β-1,3-glucan	α-1,3-glucan
Percentage (mol%)	Gal*f*	$Mn^{1,2}$	$Mn^{1,6}$	Gal*p*	GalN	GalNAc	42±4	10±1
	33±3	7.0±0.8	8±2	UD	UD	UD		

The areas of the well-resolved peaks derived from 2D ^{13}C-^{13}C DP INADEQUATE spectra.
mannose ($Mn^{1,6}$): the average of C1,C2,C3,C4
mannose ($Mn^{1,2}$): the average of C1,C2,C3,C4
β-1,5-galactofuranose (Gal*f*): the average of C1,C2,C3,C4
galactopyranose (Gal*p*): the average of C1,C2,C3,C4
galactosamine (GalN): the average of C1,C2,C3,C4
N-acetylgalactosamine (GalNAc): the average of C1,C2,C3,C4
α-1,3-glucan: the average of C1,C2,C5 and C6
β-1,3-glucan: the average of C1,C2,C3,C4

Supplementary Table 5. NMR peaks used for compositional analysis. Composition analysis were carried out using 2D 53-ms CORD and 2D J-INADEQUATE for rigid and mobile potions, respectively. Listed cross peaks (for CORD) or peak pairs (for INADEQUATE) were used for compositiona analysis.

**Rigid Phase**			
Molecule	Cross peak		
	F1 (ppm)	F2 (ppm)	Assignment
α-1,3-glucan	101.0	69.5	C1-C4
	101.0	71.9	C1-C2
	101.0	71.7	C1-C5
	84.6	71.9	C3-C2
	84.6	71.7	C3-C5
	84.6	69.5	C3-C4
β-1,3-glucan	103.6	86.4	C1-C3
	103.6	77.1	C1-C5
	103.6	68.7	C1-C4
	86.4	68.7	C3-C4
	86.4	77.1	C3-C5
	86.4	74.4	C3-C2
	77.1	68.7	C5-C4
	74.4	68.7	C2-C4
Chitin	103.6	83.0	C1-C4
	103.6	75.7	C1-C5
	103.6	55.5	C1-C2
	103.6	72.9	C1-C3
	83.0	55.5	C4-C2
	83.0	72.9	C4-C3
	83.0	75.7	C4-C5
	75.7	72.9	C5-C3
	72.9	55.5	C3-C2
	75.7	55.5	C5-C2

**Mobile Phase**		
Molecule/unit	Spin connection	
	Chemical shifts (ppm)	Assignment
α-1,6-mannose	102.7, 70.6	C1,C2
	73.2, 67.9	C3,C4
α-1,2-mannose	101.3, 78.7	C1,C2
	67.7, 73.9	C4,C5
Galactofuranose	107.5, 81.6	C1,C2
	77.7, 83.5	C3,C4
Galactopyranose	72.2, 70.7	C2,C3
	73.5, 72.5	C4,C5
Galactosamine	91.7, 54.8	C1,C2
	71.1, 81.1	C3,C4
N-acetylgalactosamine	95.7, 57.5	C1,C2
	75.2, 76.9	C3,C4
α-1,3-glucan	101.0, 71.9	C1,C2
	69.5, 71.7	C4,C5
β-1,3-glucan	103.6, 74.4	C1,C2
	68.7, 77.1	C4,C5

Supplementary Table 6. Polysaccharide composition from ssNMR data. The results are presented for the mobile and rigid phases of the alkali-soluble and alkali-insoluble fractions of parental *A. fumigatus*. UD: undetected. GAG is not detected using the AI and AS samples.

Component	**AI** (mole%)		**AS** (mole%)	
	Rigid	mobile	Rigid	mobile
β-1,3-glucan	49 ± 9	41 ± 15	UD	UD
α-1,3-glucan	14 ± 3	16 ± 4	100 ± 30	14 ± 4
chitin	23 ± 4	13 ± 3	UD	UD
GM	UD	30 ± 10	UD	78 ± 26
Amino acid (valine)	14 ± 3	UD	UD	8 ± 2

Supplementary Table 7. Chemical analysis of *A. fumigatus* polysaccharides. The alkali-soluble and alkali-insoluble fractions of the parental samples are analyzed using GC-MS methods coupled with Enzymatic degradation and HPLC purification. The samples prepared using the minimal medium were the ones characterized by NMR in this study.

Component	Minimal medium	
	**AI**	**AS**
β-1,3 glucan	47	13
α-1,3 glucan	11	55
chitin	22	1
GM	7	4
GAG	1	9

The chemical data of the sample prepared using minimal medium is in general agreement with the NMR results presented in **Supplementary Table 6**.

Supplementary Table 8. **^{13}C-T$_1$ relaxation times of major polysaccharides in wild-type *A. fumigatus*.** A single exponential equation is used to fit the data: $I(t)=e^{-t/T1}$. Error bars are standard deviations of the fit parameters.

Component	Cross-peaks	T₁ (s)		
		Parental	α-1,3-glucan def.	Chitin def.
β-1,3-glucan	B1-3	1.8±0.6	1.4±0.4	1.7±0.4
	B1-5	1.4±0.5	1.4±0.1	1.5±0.4
	B1-2	1.2±0.2	1.8±0.2	2.7±0.5
	B1-4	2.6±0.6	1.47±0.06	5.0±0.7
	B3-1	0.5±0.1	1.18±0.08	1.8±0.4
	B3-5	1.0±0.4	1.1±0.1	1.65±0.04
	B3-2	1.1±0.2	1.22±0.07	1.9±0.4
	B5-1	1.3±0.2	0.88±0.06	0.8±0.2
	B5-3	4±2	1.00±0.07	1.0±0.2
	B5.2	1.2±0.2	0.9±0.1	0.8±0.1
	B5-4	0.2±0.1	0.7±0.2	0.46±0.07
	B2-1	1.4±0.3	1.5±0.1	1.9±0.7
	B2-3	1.4±0.2	1.23±0.04	1.4±0.4
	B2-5	1.1±0.3	1.14±0.09	1.6±0.3
	B2-4	0.05±0.02	1.37±0.09	3.4±0.9
	B4-1	0.7±0.2	1.3±0.2	5.5±0.4
	B4-3	1.6±0.2	1.2±0.1	1.4±0.4
	B4-5	0.8±0.3	1.15±0.07	1.6±0.3
	B4-2	1.7±0.2	1.4±0.2	3.4±0.9
	**Average**	**1.3**	**1.2**	**2.1**
α-1,3-glucan	A1-2/5	4.2±0.9	-	5.2±0.7
	A2/5-1	2.9±0.6	-	3.9±0.9
	A2/5-4	1.9±0.7	-	5.1±0.2
	A3-1	4.3±0.8	-	4.0±0.9
	A3-2/5	3.4±0.6	-	2.6±0.6
	A4-2/5	2.9±0.9	-	3.9±0.8
	**Average**	**3.3**	**-**	**4.1**
Chitin	Ch1-4	5±1	3.5±0.9	-
	Ch1-5	2.2±0.4	1.8±0.5	-
	Ch1-3	1.2±0.2	0.8±0.4	-
	Ch3-5	1.4±0.6	4±1	-
	Ch3-1	1.9±0.1	5±2	-
	Ch5-4	2.9±0.8	3.6±0.7	-
	Ch5-3	0.8±0.4	2.4±0.5	-
	Ch5-2	2.9±0.2	3.3±0.8	-
	Ch4-5	2.8±0.4	4.4±0.8	-
	Ch2-1	1.1±0.4	5.9±0.8	-
	Ch2-4	2.9±0.4	4.6±0.6	-
	Ch2-5	1.2±0.4	3.2±0.8	-
	Ch2-3	1.3±0.3	2.7±0.7	-
	**Average**	**2.1**	**3.5**	**-**

Supplementary Table 9. Water-edited buildup curves of major polysaccharides. The data are fit using exponential growth equation: $I(t) = 1\text{-}Ae^{-t/T}$, where the prefactor accounts for the initial residual intensity. Error bars are standard deviations of the fit parameters.

Sample type	Assignment	ppm (^{13}C)	Prefactor	$\sqrt{Buildup\ time}$ ($\sqrt{ms}$)
Parental	A1	101.0	0.78	3.8 ± 0.9
	A3	84.6	0.92	3.5 ± 0.6
	A4	69.5	0.80	1.4 ± 0.2
	A2/5	71.9	0.71	1.6 ± 0.2
	B1	103.6	0.88	1.6 ± 0.3
	B2	74.4	0.83	1.6 ± 0.3
	B3	86.4	0.91	1.7 ± 0.5
	B4	68.7	0.81	1.7 ± 0.4
	B5	77.1	0.80	1.8 ± 0.6
	Ch2	55.5	0.84	3.9 ± 0.4
	Ch3	72.9	0.79	2.1 ± 0.3
	Ch4	83.0	0.86	2.2 ± 0.6
	Ch5	75.7	0.88	1.9 ± 0.4
GM def.	A1	101.0	0.88	4.6 ± 0.5
	A3	84.6	0.91	4.8 ± 0.8
	A4	69.5	0.93	3.8 ± 0.4
	A2/5	71.9	0.85	3.9 ± 0.5
	B1	103.6	0.87	4.0 ± 0.5
	B2	74.4	0.88	3.2 ± 0.4
	B3	86.4	0.92	4.0 ± 0.5
	B4	68.7	0.93	3.5 ± 0.4
	B5	77.1	0.83	2.5 ± 0.3
	Ch2	55.5	0.80	5.4 ± 0.9
	Ch3	72.9	0.87	3.9 ± 0.5
	Ch4	83.0	0.75	3.8 ± 0.6
	Ch5	75.7	0.90	3.1 ± 0.3
GAG def.	A1	101.0	0.92	4.8 ± 0.8
	A3	84.6	0.92	4.6 ± 0.7
	A4	69.5	0.89	3.3 ± 0.4
	A2/5	71.9	0.89	4.1 ± 0.5
	B1	103.6	0.94	2.8 ± 0.3
	B2	74.4	0.83	3.4 ± 0.4
	B3	86.4	0.83	3.2 ± 0.5
	B4	68.7	0.85	3.0 ± 0.4
	B5	77.1	0.83	3.1 ± 0.3
	Ch2	55.5	0.90	4.0 ± 0.8
	Ch3	72.9	0.93	5.5 ± 0.6
	Ch4	83.0	0.98	4.5 ± 0.5
	Ch5	75.7	0.85	3.9 ± 0.4

Chitin def.	A1	101.0	0.91	4.1 ± 0.6
	A3	84.6	0.94	4.1 ± 0.5
	A4	69.5	0.91	3.1± 0.3
	A2/5	71.9	0.88	3.5 ± 0.4
	B1	103.6	0.88	2.7 ± 0.3
	B2	74.4	0.86	2.6 ± 0.3
	B3	86.4	0.93	3.1 ± 0.4
	B4	68.7	0.92	2.8 ± 0.3
	B5	77.1	0.86	2.5 ± 0.3
α-1,3- glucan def.	B1	103.6	0.93	2.8 ± 0.3
	B2	74.4	0.91	2.9 ± 0.3
	B3	86.4	0.96	2.6 ± 0.3
	B4	68.7	0.90	2.7 ± 0.3
	B5	77.1	0.90	2.8 ± 0.3
	Ch2	55.5	0.96	5.1 ± 0.9
	Ch3	72.9	0.95	3.0 ± 0.4
	Ch4	83.0	0.98	4.3 ± 0.4
	Ch5	75.7	0.93	3.0 ± 0.4

Supplementary References

1. Shim, J. H.; Sung, K. J.; Cho, M. C.; Choi, W. A.; Yang, Y.; Lim, J. S.; Yoon, D. Y., Antitumor effect of soluble beta-1,3-glucan from Agrobacterium sp. R259 KCTC 1019. *J. Microbiol. Biotechnol.* **2007,** 17, (9), 1513-1520.
2. Fairweather, J. K.; Him, J. L.; Heux, L.; Driguez, H.; Bulone, V., Structural characterization by (13)C-NMR spectroscopy of products synthesized in vitro by polysaccharide synthases using (13)C-enriched glycosyl donors: application to a UDP-glucose:(1-->3)-beta-D-glucan synthase from blackberry (Rubus fruticosus). *Glycobiology* **2004,** 14, (9), 775-81.
3. Saitô, H.; Ohki, T.; Sasaki, T., A 13C-nuclear magnetic resonance study of polysaccharide gels. Molecular architecture in the gels consisting of fungal, branched (1 → 3)-β-d-glucans (lentinan and schizophyllan) as manifested by conformational changes induced by sodium hydroxide. *Carbohydr. Res.* **1979,** 74, (1), 227-240.
4. Bhanja, S. K.; Rut, D.; Patra, P.; Sen, I. K.; Nandan, C. K.; Islam, S. S., Water-insoluble glucans from the edible fungus Ramaria botrytis. *Bioactive Carbohydrates and Dietary Fibre* **2014,** 3, (2), 52-58.
5. Puanglek, S.; Kimura, S.; Enomoto-Rogers, Y.; Kabe, T.; Yoshida, M.; Wada, M.; Iwata, T., In vitro synthesis of linear alpha-1,3-glucan and chemical modification to ester derivatives exhibiting outstanding thermal properties. *Sci. Rep.* **2016,** 6.
6. Kono, H.; Numata, Y.; Erata, T.; Takai, M., ^{13}C and ^{1}H resonance assignment of mercerized cellulose II by two-dimensional MAS NMR spectroscopies. *Macromolecules* **2004,** 37, (14), 5310-5316.
7. Heux, L.; Brugnerotto, J.; Desbrieres, J.; Versali, M. F.; Rinaudo, M., Solid state NMR for determination of degree of acetylation of chitin and chitosan. *Biomacromolecules* **2000,** 1, (4), 746-751.
8. Kameda, T.; Miyazawa, M.; Ono, H.; Yoshida, M., Hydrogen bonding structure and stability of alpha-chitin studied by C-13 solid-state NMR. *Macromol. Biosci.* **2004,** 5, (2), 103-106.
9. King, C.; Stein, R. S.; Shamshina, J. L.; Rogers, R. D., Measuring the Purity of Chitin with a Clean, Quantitative Solid-State NMR Method. *ACS Sustain. Chem. Eng.* **2017,** 5, (9), 8011-8016.
10. Tanner, S. F.; Chanzy, H.; Vincendon, M.; Roux, J. C.; Gaill, F., High-Resolution Solid-State C-13 Nuclear-Magnetic-Resonance Study of Chitin. *Macromolecules* **1990,** 23, (15), 3576-3583.
11. Latgé, J. P.; Kobayashi, H.; Debeaupuis, J. P.; Diaquin, M.; Sarfati, J.; Wieruszeski, J. M.; Parra, E.; Bouchara, J. P.; Fournet, B., Chemical and immunological characterization of the extracellular galactomannan of Aspergillus fumigatus. *Infection and immunity* **1994,** 62, (12), 5424-33.
12. Fontaine, T.; Delangle, A.; Simenel, C.; Coddeville, B.; van Vliet, S. J.; van Kooyk, Y.; Bozza, S.; Moretti, S.; Schwarz, F.; Trichot, C.; Aebi, M.; Delepierre, M.; Elbim, C.; Romani, L.; Latgé, J. P., Galactosaminogalactan, a new immunosuppressive polysaccharide of Aspergillus fumigatus. *PLoS pathogens* **2011,** 7, (11), e1002372.
13. K J Fritzsching; Y Yang; K Schmidt-Rohr; Mei Hong, Practical use of chemical shift databases for protein solid-state NMR: 2D chemical shift maps and amino-acid assignment with secondary-structure information. *J Biomol NMR.* **2013,** 56.
